# Confidence in Phase Definition for Periodicity in Genes Expression Time Series

**DOI:** 10.1371/journal.pone.0131111

**Published:** 2015-07-10

**Authors:** Mohammed El Anbari, Abeer Fadda, Andrey Ptitsyn

**Affiliations:** Division of Biomedical Informatics, Sidra Medical and Research Center, Doha, Qatar; University of Georgia, UNITED STATES

## Abstract

Circadian oscillation in baseline gene expression plays an important role in the regulation of multiple cellular processes. Most of the knowledge of circadian gene expression is based on studies measuring gene expression over time. Our ability to dissect molecular events in time is determined by the sampling frequency of such experiments. However, the real peaks of gene activity can be at any time on or between the time points at which samples are collected. Thus, some genes with a peak activity near the observation point have their phase of oscillation detected with better precision then those which peak between observation time points. Separating genes for which we can confidently identify peak activity from ambiguous genes can improve the analysis of time series gene expression. In this study we propose a new statistical method to quantify the phase confidence of circadian genes. The numerical performance of the proposed method has been tested using three real gene expression data sets.

## Introduction

Analysis of periodic patterns is an essential part of many studies of gene expression involving timeline sampling or targeting of rhythmically expressed genes. Recent publications report a large proportion of the entire transcriptome oscillating in a circadian (i.e. approximately daily) rhythm [1–3]. The number of genes for which circadian baseline can be identified as statistically significant over stochastic noise is traditionally thought to be under 10% [4–6], but more recently estimated as 43% [1] or even close to 100% [7], depending on the algorithms applied. Significance of the signal-to-noise ratio is the focus of most studies targeting rhythmic expression. The absolute amplitude and time of the peak (i.e. phase) of rhythmic gene expression are also analyzed and reported. However, we feel that one aspect of rhythmic gene expression required additional consideration. It has been observed that low sampling frequency presents a significant challenge to all studies of periodic gene expression ([7] for review). Most gene expression studies only report 6 or 9 observation points per period and not more than two consecutive periods in the entire timeline. Some oscillating genes may have peak expression coinciding at, or near, the observation point (i.e. the time when the animal is sacrificed and tissue samples are taken for analysis). However, other genes may peak at any time between sparsely placed observations. Since our ability to differentiate events in time is restricted by the low sampling rate, how can we be sure that genes are expressed in the phase we identified? Would it be possible to make a quantitative estimation of confidence that a gene peaks at a certain time of the day? With such a metric we could separate a fraction of genes for which we know the true time of peak and analyze the function of genes at a given time with less noise (i.e. genes highly expressed, but peaking at a different time) mixed in. To answer these questions and enable time-wise analysis of gene function and interactions, we propose a novel algorithm for the estimation of confidence of phase assignment in analysis to timeline expression profiles.

To answer the questions posed for this study we propose to use the bootstrap, which is a general technique for estimating unknown quantities associated with statistical models. Often the bootstrap is used to find

- standard errors for estimators,
- confidence intervals for unknown parameters,
- *p*-values for test statistics under a null hypothesis.

The maximum entropy bootstrap [8] is a resampling method for observations that are not necessarily independent and/or identically distributed. These conditions match typical observations of gene expressions time series. The maximum entropy bootstrap is an algorithm that constructs a large number of replicates (such as *R* = 999) that retain the basic shape, local peaks and troughs and time independence of the original time series, by being strongly dependent on it. The maximum entropy bootstrap is particularly useful for short time series.

## Materials and Methods

### Notations

I: indicator function.
*n*: the sample size.
*p*: the number of genes.
*χ* = {*X*_1_, …, *X*_*n*_}: random sample from population.
$\chi^{*}=\{X_{1}^{*},...,X_{n}^{*}\}$: resample obtained by sampling from *χ*.
*α*: level of confidence.
$\hat{\theta }$: estimate of *θ*, computed from *χ*.
${\hat{\theta }}^{*}$: bootstrap version of $\hat{\theta }$, computed from χ*.

### Phase estimation

We consider a gene expression time series {*x*_1_, …, *x*_*n*_}. Without loss of generality, suppose that the measurements are taken in time points *t* = 0, 4, 8, …, 44*h*. We can then construct a collection of intervals named *phases* and labeled *G*_0_, *G*_1_, …, *G*_5_ such that

$$\begin{array}{c} G_{0}=\left[-2,2\right],G_{1}=\left[2,6\right],G_{2}=\left[6,10\right],G_{3}=\left[10,14\right],G_{4}=\left[14,18\right],G_{5}=\left[18,22\right]. \end{array}$$ (1)

Let *θ* be the first *peak time* or the *phase* of the gene expression. We are interested in *estimating* and in a later step *constructing a confidence interval* for *θ*. More precisely, we want to construct an interval contained in one of the classes *G*_*i*_, and that contains the estimated parameter $\hat{\theta }$ with high probability.

**Fig 1:**
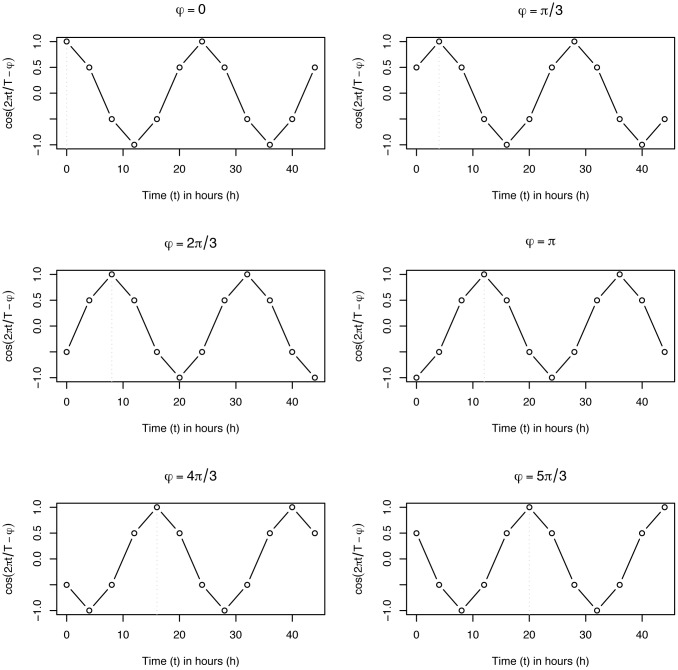
Graph of the ideal cosines. Graph representing the cosine waves: $\text{cos}(\frac{2\pi }{T}t-\varphi )$ for *t* = 0,4,8, …, 44 and *φ* ∈ {0, *π*/3, 2*π*/3, *π*, 5*π*/3}. The dotted vertical line shows the first *peak time*.

The expression profile of a gene exhibiting circadian rhythmicity approximates to a cosine wave with a period *T* = 24h. A significant correlation can therefore be found between rhythmically expressed gene and a theoretical cosine wave cycling with an appropriate phase. The process of estimating *θ* consists of the following steps:

1. Generate 6 cosine waves with the equation given below
  $$\begin{array}{c} {𝒞}_{\varphi }\left(t\right)=\cos\left(\frac{2\pi }{T}t-\varphi \right),t=0,4,8,...,44;\varphi \in \left\{0,\pi /3,2\pi /3,\pi ,4\pi /3,5\pi /3\right\}. \end{array}$$ (2)
  The following properties apply: the periods are 24h, 48h long (two cycles), and the intervals between adjacent phases is 4h. Fig 1 is a graphical representation of the cosine Eq (2).
2. Calculate the correlation coefficient between the gene expression profile and each of the 6 cosine waves 𝒞_*φ*_. Let $R=\{{\hat{\rho }}_{0},{\hat{\rho }}_{1},...,{\hat{\rho }}_{5}\}$ denotes the obtained vector of correlations. Let
  $$\begin{array}{c} \hat{\rho }=\max R, \end{array}$$ (3)
  be the highest correlation and $\hat{\varphi }$ the phase of the corresponding cosine wave. The optimal ${𝒞}_{\hat{\varphi }}$ is selected to be the representative of the circadian rhythmicity if the correlation is significant. Our parameter of interest *θ* is then estimated by the peak of the *best-correlated* cosine curve, and it is equal to
  $$\begin{array}{c} \hat{\theta }=\hat{\varphi }T/2\pi =12\hat{\varphi }/\pi . \end{array}$$ (4)

### Data resampling using Maximum Entropy Bootstrap Algorithm

**Fig 2:**
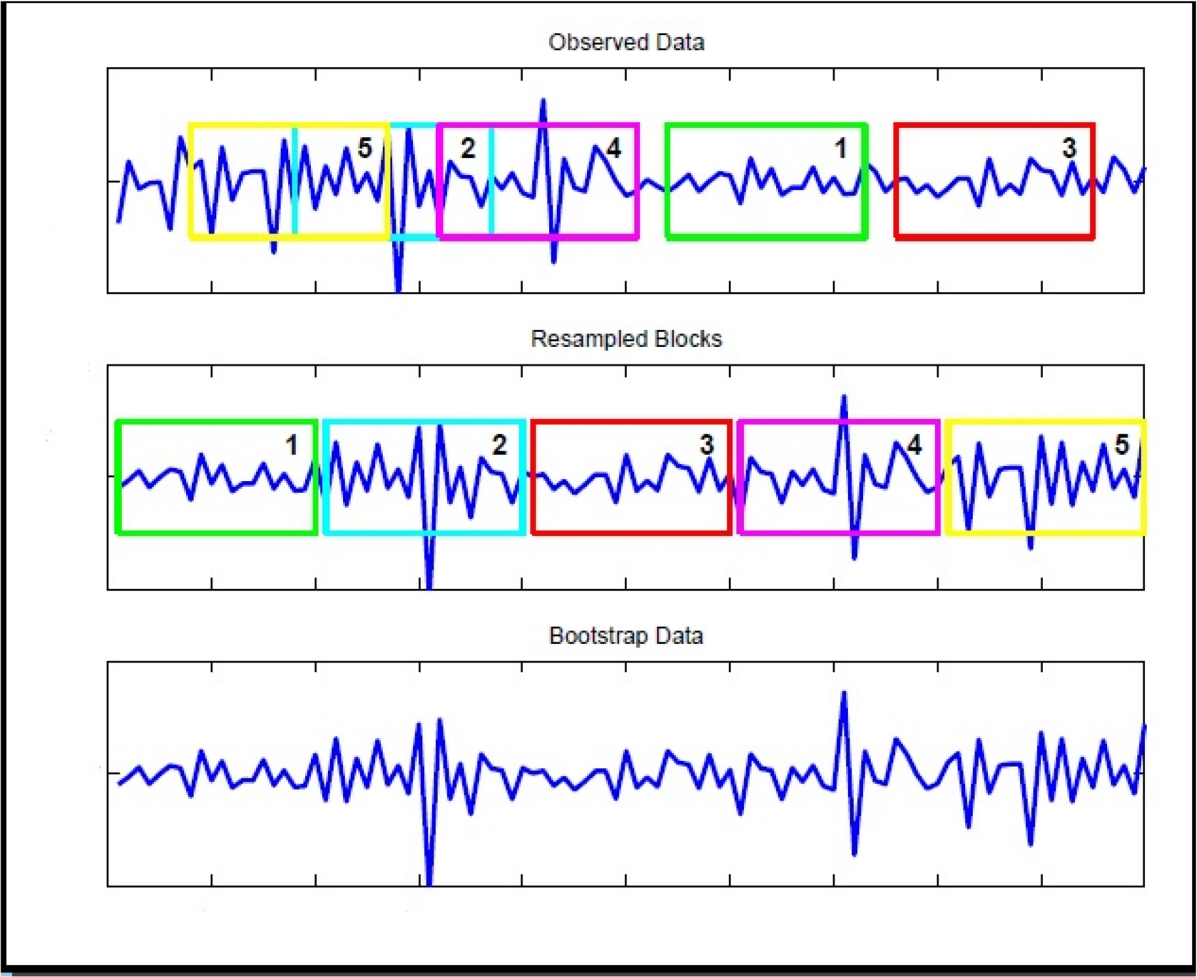
Graph of the moving block bootstrap principle. Graph showing the principal of moving block bootstrap. The moving block bootstrap randomly selects blocks of the original data (top) and concatenate them together (center) to form a resample (bottom).

Several bootstrap methods have been proposed for time series data. The most well-known is the *Moving Block Bootstrap*. This procedure works by dividing the observations in blocks of length *b* and then resampling the blocks (See Fig 2 for an illustration). The main problem with the block bootstrap is that the block length, *b*, which is a form of smoothing parameter, needs to be chosen. If the blocks are too short, the bootstrap samples cannot mimic the original sample. In this case dependency is broken whenever we start a new block. If, on the other hand, the blocks are too long, we will lose the randomness of the replicates. For these reasons, in this study we apply the maximum entropy bootstrap algorithm proposed by [8]. It does not impose strong assumptions on the distribution of the time series like stationarity. A full description of the algorithm can be found in [9]. The replications are generated by the following steps

1. Form order statistics *x*_(*t*)_ by sorting increasingly the original data, and keep the vector of ordering index.
2. Using the ordering statistics obtained at step 1, compute the intermediate points *z*_(*t*)_ = (*x*_(*t*)_ + *x*_(*t* + 1)_)/2 for *t* = 1, …, *n* − 1.
3. For *t* = 1, …, *n*, construct the deviation *x*_(*t*)_ − *x*_(*t* − 1)_, and calculate the trimmed mean *m*_trm_ of the obtained observations. The lower limit for left tail is *z*_0_ = *x*_(1)_ − *m*_trm_ and upper limit for right tail is *z*_*n*_ = *x*_(*n*)_ + *m*_trm_. *z*_0_ and *z*_*n*_ are the new limiting intermediate points.
4. Compute the mean of the maximum entropy (ME) density within each interval while satisfying the *mean-preserving constraint*.
5. Generate uniformly distributed numbers on the [0, 1] interval, then calculate sample quantiles of the Maximum Entropy at the generated points and sort them.
6. Using the ordering index of step 1, reorder the sorted sample. This process permits to conserve the dependance relationships among observations in the original data.
7. The steps 2 to 6 are repeated many times, in our analysis we use *R* = 999.

**Fig 3:**
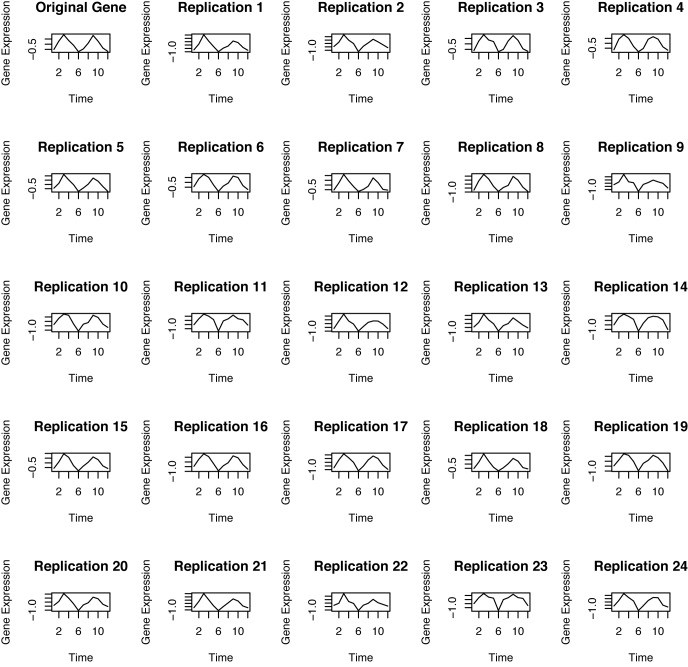
An Example of data resampling using the Maximum Entropy Bootstrap Algorithm. (Top left panel): A gene expression time series from the IWAT data. (Remaining:) Set of 24 replications randomly chosen from 999 maximum entropy bootstrap samples used in the analysis.

A complete simulated example for illustration of each step of the algorithm can be found in [9]. Fig 3 shows one gene expression time series from the IWAT data, along with 24 different replicates of the series chosen randomly from 999 used in the analysis. Due to the fact that the maximum entropy algorithm tries to retain all the properties of the data, one can see that the replicates remain close to the original time series.

### The Bootstrap Approach for *p*-value

Let $\hat{\tau }$ denote the realized value of a test statistic *τ* computed for a particular sample. Then $\mathbb{P}(\tau \geq \hat{\tau }|H_{0})$ is the definition of the *p*-value in situations where large values of *τ* support the alternative hypothesis. The process of calculating *p*-value consists of the following steps:

1. Specify a way to generate bootstrap samples that resemble the real data while satisfying the null hypothesis *H*_0_. In our case we will use the **Maximum Entropy Bootstrap Algorithm**.
2. Let **MEBA** denote this **bootstrap data-generating process**.
3. Using **MEBA**, generate *R* = 999 bootstrap samples indexed by *j*. From each of them, compute a bootstrap test statistic $\tau_{j}^{*}$. To estimate a bootstrap *p*-value, we use
  $$\begin{array}{c} {\hat{p}}^{*}\left(\hat{\tau }\right)=\frac{1+\sum_{j=1}^{R}I_{\left\{\tau_{j}^{*}>\hat{\tau }\right\}}}{1+R}. \end{array}$$ (5)
  Arguments in favor of the latter formulae for calculating *p*-value instead of the classical formulae $\sum_{j=1}^{R}{\text{I}}_{\left\{\tau_{j}^{*}>\hat{\tau }\right\}}/R$, can be found in [10], p. 148, 161). For example, if 73 of the $\tau_{j}^{*}$ are greater than $\hat{\tau }$, then ${\hat{p}}^{*}(\hat{\tau })=(1+73)/(1+999)=0.074$.
4. Reject the null hypothesis *H*_0_ if ${\hat{p}}^{*}(\hat{\tau })<\alpha$. Where *α* is a given constant satisfying 0 < *α* < 1. In general we take *α* = 0.05.

This algorithm will be used to assess significance of the correlation between a gene expression time series and one of the cosine Eq (2).

### Bootstrap Percentile Confidence Interval

The main focus of this paper is to give an accurate approximate confidence interval for *peak time* parameter $\hat{\theta }$. Computing such confidence intervals with distributions that are difficult to represent mathematically, is very challenging. The bootstrap is another class of general methods for constructing confidence intervals without making strong distributional assumptions about the data or the statistic being calculated. There are several ways to construct bootstrap confidence intervals. They vary in ease of calculation and accuracy. There have been three main lines of development: Efron’s original percentile method [11], the bootstrap *t* interval introduced in [12], and the double bootstrap interval introduced in [13]. In this work, due to its simplicity and good performance, we use the Bootstrap Percentile Confidence Interval.

Let $\hat{\theta }$ be an estimator of *θ* on the measured data *X*_1_, …, *X*_*n*_, and ${\hat{\theta }}^{*}$ be its analog on a bootstrapped sample $X_{1}^{*},...,X_{n}^{*}$, then:

$$\begin{array}{c} K_{\text{boot}}\left(x\right)={\mathbb{P}}_{*}\left(\left\{{\hat{\theta }}^{*}\leq x\right\}\right). \end{array}$$ (6)

Where *K*_boot_ is the empirical distribution function of the bootstrap values. Efron’s (1979) original 100(1 − 2*α*)% bootstrap *percentile interval* is to just take the empirical 100*α* and 100(1 − *α*) percentiles from the bootstrap values ${\hat{\theta }}_{1}^{*},...,{\hat{\theta }}_{R}^{*}$. Then the 100(1 − 2*α*)% percentile interval is

$$\begin{array}{c} \left[{\underline{\theta }}_{\text{bp}},{\bar{\theta }}_{\text{bp}}\right]=\left[K_{\text{boot}}^{-1}\left(\alpha \right),K_{\text{boot}}^{-1}\left(1-\alpha \right)\right], \end{array}$$ (7)

where $K_{\text{boot}}^{-1}$ is the inverse or the generalized inverse distribution function or quantile function. The name percentile comes from the fact that $K_{\text{boot}}^{-1}(\alpha )$ and $K_{\text{boot}}^{-1}(1-\alpha )$ are percentiles of the bootstrap distribution *K*_boot_ in Eq (6). In practice, we proceed as follows:

1. Generate *R* bootstrap samples of size *n* using the maximum entropy algorithm.
2. Estimate the parameter *θ* of interest for each bootstrap sample: ${\hat{\theta }}_{b}^{*}$ for *b* = 1, …, *R*.
3. Order the bootstrap replications of $\hat{\theta }$ such that ${\hat{\theta }}_{\left(1\right)}^{*}\leq {\hat{\theta }}_{\left(2\right)}^{*}\leq ...{\hat{\theta }}_{\left(R\right)}^{*}$. The lower and upper confidence bounds are the *R*.*α* ^th^ and *R*.(1 − *α*)^th^ ordered elements, respectively. The estimated (1 − 2*α*) confidence interval of $\hat{\theta }$ is
  $$\begin{array}{c} \left[{\underline{\theta }}_{\text{bp}},{\bar{\theta }}_{\text{bp}}\right]=\left[{\hat{\theta }}_{\left(R.\alpha \right)}^{*},{\hat{\theta }}_{\left(R.(1-\alpha )\right)}^{*}\right]. \end{array}$$ (8)

**Fig 4:**
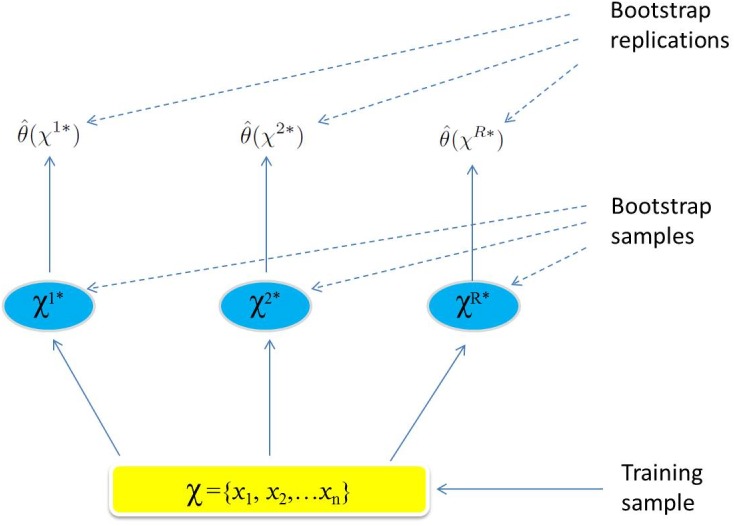
The Bootstrap Percentile confidence interval principle. Schematic of the bootstrap process. We want to estimate a confidence interval for the phase *θ*(*χ*). *R* training sets, *χ* ^1^*, …, *χ* ^R^* each of size *n* are generated using an appropriate resampling mechanism. The quantity of interest *θ*(*χ*) is computed from each bootstrap training set, and the values $\theta (\chi_{1}^{*}),...,\theta (\chi_{R}^{*})$ are used to construct a confidence interval for the quantity *θ*(*χ*).

Fig 4 summarizes the steps of the Bootstrap Percentile confidence interval principle.

**Remark 1**. If *R*.*α* is not an integer, the following procedure can be used:

Let *k* = [(*R* + 1)*α*], the largest integer ≤ (*R* + 1)*α*. Then we define the empirical *α* and (1 − *α*) quantities by the *k* ^th^ largest and (*R* + *k* − 1)^th^ values of ${\hat{\theta }}_{\left(b\right)}^{*}$, respectively. So if *R* = 999 and *α* = 2.5% these are the 25^th^ and 975^th^ ordered elements.

We have now all the pieces needed to accomplish the phase confidence analysis. Algorithm 1 summarizes the details of the proposed approach

**Algorithm 1**: Confidence in phase definition for periodicity in genes expression time series

**Data**: *χ* = {*x*_1_, …, *x*_*n*_}: *n* realizations of a gene expression time series, the number of replications *R*, and a confidence level *α*.

**Result**: Bootstrapped *p*-value, Bootstrap Percentile Confidence Interval $\left[{\underline{\theta }}_{\text{bp}},{\bar{\theta }}_{\text{bp}}\right]$.

1 **for** *b* ← 1 **to** *R* **do**

2    Using the maximum entropy bootstrap algorithm, generate a bootstrap sample *χ* ^b*^;

3    Calculate the maximum correlation ${\hat{\rho }}_{b}$ using formula Eq (3);

4    Estimate the peak time ${\hat{\theta }}_{b}$ using formula Eq (4);

5 Calculate the bootstrapped *p*-value ${\hat{p}}^{*}(\hat{\rho })$ using Eq (5);

6 **if** ${\hat{p}}^{*}(\hat{\rho })\leq \alpha$ **then**

7    the gene is considered as circadian.

8 Calculate the Bootstrap Percentile Confidence Interval $\left[{\underline{\theta }}_{\text{bp}},{\bar{\theta }}_{\text{bp}}\right]$ using Eq (8).

9 **if** *it exist* *i* ∈ {0, …, 5} such that $[{\underline{\theta }}_{\text{bp}},{\bar{\theta }}_{\text{bp}}]\subset G_{i}$ **then**

10  the gene is assigned to the phase *G*_*i*_, where *G*_*j*_ are defined in Eq (1).

## Results, Discussion, and Conclusions

We conducted experiments on three real previously published data sets. The data are derived from microarray study of gene expression in three tissues in mice referred as Inguinal White Adipose tissue (IWAT), Brown Adipose Tissue (BAT) and Liver. Each individual data set contains more than 22,000 gene expression profiles. Each profile consists of 12 time points of 4-h interval difference. See [14] for detailed description. In the first step of our analysis, we estimated the phase of each gene using the Eq (4), and we identified the circadian gene expression based on the Algorithm 1. We note here that our aim is not to identify all the circadian genes, but we are more interested in genes for which the peak time is near to one of the time points where the measurements are taken. Detection of circadian genes can be sophistically performed using Fisher’s *g*-test, autocorrelation or permutation test (See [15] for more details). This estimation revealed 646 oscillatory genes in the IWAT data, 680 in the BAT data, and 747 in the Liver data for which the bootstrapped *p*-value was ≤ 0.05, representing 6.9%, 7.15%, and 7.6% of the number of oscillatory genes obtained by applying a permutation test, respectively.

We used our proposed method to calculate a 95% confidence interval $\left[{\underline{\theta }}_{\text{bp}},{\bar{\theta }}_{\text{bp}}\right]$ for the *peak time* of the oscillating genes, and then we assigned a phase to each of them using the following rule: a circadian gene is assigned to a Phase *G*_*i*_ if $[{\underline{\theta }}_{\text{bp}},{\bar{\theta }}_{\text{bp}}]\subset G_{i}$.

**Fig 5:**
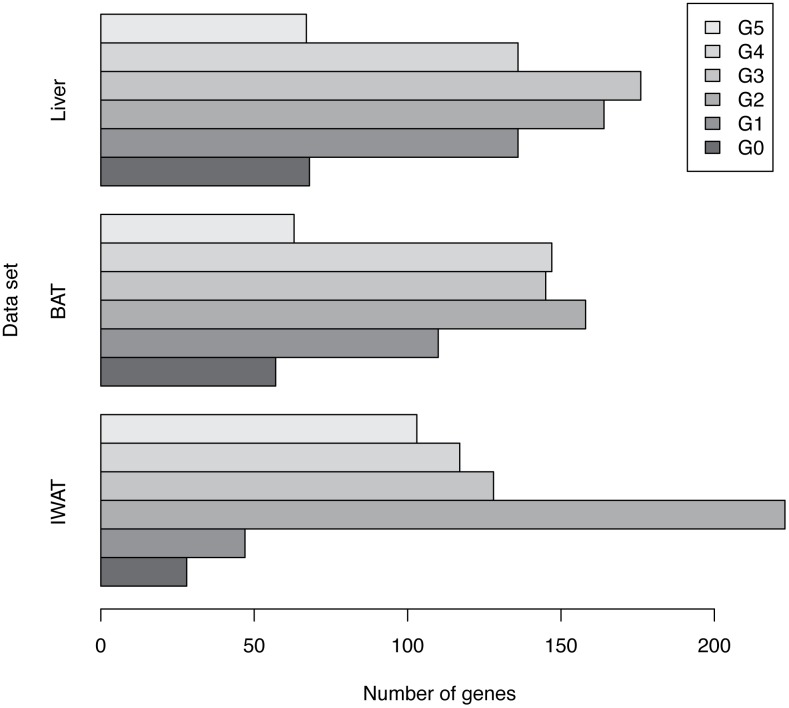
Barplot of the number of genes against phases. Bar plot summarizing the number of genes in each phase for the IWAT, BAT and Liver data sets from the results in Table 1.

The Results of phase classification are summarized in Table 1 and Fig 5. In the IWAT data, and with a confidence levels of at least 95%, 28 genes peak at Phase *G*_0_, 47 at Phase *G*_1_, 223 at Phase *G*_2_, 128 at Phase *G*_3_, 117 at Phase *G*_4_, and 103 at Phase *G*_5_, representing 4.33%, 7.27%, 34.52%, 19.81%, 18.11%, and 15.94% of the oscillating genes, respectively. In the BAT data, 57 peak at Phase *G*_0_, 110 at Phase *G*_1_, 158 at Phase *G*_2_, 145 at Phase *G*_3_, 147 at Phase *G*_4_, and 63 at Phase *G*_5_, representing 8.38%, 16.17%, 23.23%, 21.32%, 21.61%, and 9.26% of the oscillating genes, respectively. For the Liver data set, 68 genes peak at Phase *G*_0_, 136 at Phase *G*_1_, 164 at Phase *G*_2_, 176 at Phase *G*_3_, 136 at Phase *G*_4_, and 67 at Phase *G*_5_, representing 9.10%, 18.20%, 21.95%, 23.56%, 18.20%, and 8.97% of the oscillating genes, respectively. The method for estimation of phase assignment confidence that we proposed allows some useful observation even on the testing data. For instance, we may ask how uniform is gene expression over time? For the experiments collecting data in circadian timeline we can formulate the Null-hypothesis stating that the same number of genes can be confidently assigned to each phase group. The alternative hypothesis would state that at least one phase group has significantly different number of genes. Both hypotheses are consistent with the overall number of rhythmically expressed genes and cannot be testes without quantitative estimation of confidence of phase assignment. In our test data we apply the same *p* = 0.05 threshold, but observe fewer genes peaking at one of the phases. In biological terms this means the in murine adipose tissue there is a period (morning hours) when the overall gene expression activity is lower compared to all other times of the day.

**Table 1:** Number of genes in each phase for the IWAT, BAT and Liver data sets.

Phase/Data	IWAT	BAT	Liver
Phase *G*_0_	28	57	68
Phase *G*_1_	47	110	136
Phase *G*_2_	223	158	164
Phase *G*_3_	128	145	176
Phase *G*_4_	117	147	136
Phase *G*_5_	103	63	67
**Total**	646	680	747

However, it is even more important that our method can be applied to increase precision of observation in many studies involving timeline observation of gene expression. The sampling frequency still imposes limitation on our ability to separate molecular events (such as peak of gene expression) in time. To know the time of peak expression more precisely the experiment has to be repeated with higher a number of time points (for example, one sample every 2 hours rather than every 4 hours). However, with our method we can refine the existing data. For the groups peaking at a certain time we can be confident (at a selected confidence level) that certain genes peak at a certain time and filter out genes peaking sometime between out observation time points. This confidence is essential for functional annotation of co-expressed genes and can be critical in analysis of permutation of gene activity in reaction to environment or medication.

## Strengths and boundaries

We compare the proposed method with some competing algorithms, namely Fisher’s *g*-test [16], Permutation test [15], and JTK-CYCLE [17]. All methods except the permutation test are implemented in R, and run on an Itel core *i*7 at 3.40 GHz. The permutation test is implemented in C++. Tables 2, 3 and 4 show some results for the IWAT, BAT and Liver data sets.

In this paper, we are interested in genes that may have a peak expression coinciding or near one of the observation points. We approximate their expression profiles by an ideal cosine wave of the form:

$$\begin{array}{c} {𝒞}_{\varphi }\left(t\right)=\cos\left(\frac{2\pi }{T}t-\varphi \right),t=0,4,8,...,44;\varphi \in [0,2\pi [. \end{array}$$ (9)

We know that for circadian genes we have *T* = 24h. For the data sets used in this paper, the measurements time are *t* ∈ {0,4,8, …, 44}. Since we are interested by the first peak expression time, the possible time points to be considered are *t* ∈ {0,4,8, …, 20}. If we solve for equations 𝒞_*φ*_(*t*) = 0 for *t* ∈ {0,4,8, …, 20}, we obtain *φ* ∈ {0, *π*/3, 2*π*/3, *π*, 4*π*/3, 5*π*/3}, this explains the use of *π*/3 as a resolution power of estimated phase in Eq (2). If we choose different values of the resolution power of estimated phase, the peak time of the generated ideal cosine waves will not necessarily coincide with one of the time points when the measurements were taken. Nevertheless, the method is general. It can work for periods other than 24 hours, for different spacing time points, and it can works with a larger number of cosines waves with smaller phases. For example, for any integer *k* we can generate 2*k* cosine waves using the equation:

$$\begin{array}{c} {𝒞}_{i}=\cos\left(2\pi \left(\frac{1}{24}t-\frac{1}{2k}i\right)\right)=\cos\left(\pi \left(\frac{1}{12}t-\frac{1}{k}i\right)\right),t=0,4,8,...,44;i=0,1,2,...,2k-1. \end{array}$$ (10)

Table 2 shows some timing results for *k* = 30, which generates 60 cosine waves. Results are given for *R* ∈ {9,99,999} bootstrap replications. Like any method based on resampling, the proposed method can be computationally expensive, because it involves fitting the same statistical method a large number of times using different replications of the original data. We can see that the average CPU timings increases with number of generated cosine waves and the number of bootstrap replications.

**Table 2:** IWAT, BAT and Liver data sets: timings (in minutes (m) or in hours (h)) for the proposed method and a variant of it that uses a set of 60 cosine waves with smaller phases generated using the Eq (10). The number of bootstrap replications *R* is in {9, 99, 999}.

Data set	IWAT			BAT			Liver		
Method	*R* = 9	*R* = 99	*R* = 999	*R* = 9	*R* = 99	*R* = 999	*R* = 9	*R* = 99	*R* = 999
Proposed Method using Eq (2)	3.71(m)	32.20(m)	5.53(h)	3.67(m)	32.54(m)	5.75(h)	3.61(m)	32.39(m)	5.79(h)
Proposed Method using Eq (10)	11.40(m)	1.86(h)	19.83(h)	11.39(m)	1.82(h)	19.83(h)	11.45(m)	1.81(h)	19.73(h)

Table 3 shows some timing results for the three different datasets; Fisher’s *g*-test is faster, followed by JTK-CYCLE and then the proposed method (one replication). We note here that the computing performance of the proposed method can be enhanced considerably (See **Remark 4**).

**Table 3:** IWAT, BAT and Liver data sets: timings (seconds) for Fisher’s *g*-test, Permutation test, JTK-CYCLE, and the proposed method on one bootstrap replication.

Data set	IWAT	BAT	Liver
Method			
Fisher’s *g*-test	11.79(secs)	12.27(secs)	11.87(secs)
JTK-CYCLE	16.49(secs)	14.34(secs)	14.59(secs)
Proposed Method (One replication)	22.50(secs)	22.63(secs)	22.41(secs)

Table 4 shows the number of identified circadian genes. The Permutation test identifies the highest number, followed by the JTK-CYCLE and then Fisher’s *g*-test. Our method is not developed for detecting all the circadian genes, but rather it detects, with high confidence, the circadian gene for which the peak time (the phase) is near one of the time points; estimates this phase, and constructs a confidence interval for it. This explains the small number of circadian genes detected by our method compared to the competitors.

**Table 4:** IWAT, BAT and Liver data sets: number of circadian genes identified using Fisher’s *g*-test, Permutation test and JTK-CYCLE respectively.

Data set	IWAT	BAT	Liver
Method			
Fisher’s *g*-test	4177	4547	5030
Permutation test	9321	9441	9775
JTK-CYCLE	6646	6868	7354
Proposed Method	646	680	747

**Remark 2**. This experiment design is rather typical for circadian biology. Some experiments collect samples at different intervals, such as 3h or, rarely, every 2h. Higher sampling frequency improves resolution ability, but costs a lot more and is harder to implement.

**Remark 3**. Gene expression profiles are analyzed independently, thus it is possible that a researcher may find few or none of the gene confidently peaking at a given time. In fact, in the data set on which we tested the method, gene expression has a quiet period at which relatively few genes are active.

**Remark 4**. We note that the computational performance of the proposed method can be enhanced. In fact, if we avoid using *loops* in R script that process one element per iteration, and instead we use *apply* family of functions that process whole rows, columns, or lists, the computing time is reduced significantly. In this case we need just 0.001 second to run the method for one replication using Eq (2), and we need 0.008 second to run the method using higher number of cosine waves using Eq (10).

## Supporting Information

S1 R Codes R Analysis Codes. (PDF)

S1 Data IWAT data measurements. (TXT)

S2 Data BAT data measurements. (TXT)

S3 Data Liver data measurements. (TXT)
