# Supplementary material for: Confidence in Phase Definition for Periodicity in Genes Expression Time Series
Source: PLoS One. 2015 Jul 10;10(7):e0131111. doi: 10.1371/journal.pone.0131111 (PMC4498625; doi:10.1371/journal.pone.0131111)
Supplement: S1 R Codes — (PDF) [file pone.0131111.s001.pdf]

Supplemental Materials: R codes

```
# The following function generates T data points
# from the ideal cosine waves in equations (2) for a given of phi.

ideal_cosine<-function(T,phi){
r<-cos((2*pi*(1/24)*T) - phi)
return(r)
}

# The following function generates T data points
# from the ideal cosine waves in equations (2) for all values of phi.

ideal_cos_matrix<- function(T,PHI){
mat<- matrix(0,nr=length(PHI),nc=length(T))

for(i in 1:length(PHI)){
mat[i,]<- ideal_cosine(T,PHI[i])
}
return(mat)
}

#==============Calculation of the Maximal correlation ===============
# The following function finds the most correlated ideal
# cosine with a given gene expression profile

max_cor<-function(X,T,PHI){
X<-as.numeric(X)
m<-ideal_cos_matrix(T,PHI)
dvec<-rep(0, length(PHI))
for(i in 1:length(PHI)) {dvec[i]<-cor(X, m[i,])}
ind<- which(dvec==max(dvec))
ind<-ind[1]
peakTime<-12*PHI[ind]/pi
results<-list(cor_Distances_vec=dvec,Maximal_cor_index=ind,
              Maximal_cor=dvec[ind],cor_best_cos=m[ind,],
              T=T,Maximal_cor_peakTime=peakTime)
return(results)
}

#==============Single Maximum Entropy Analysis===============
# The following function do the whole analysis for a single gene

single_meboot_analysis<-function(X,R,T,PHI){
```

```
library(meboot)
X<-as.numeric(X)
phase_vec<-rep(0,R)
cor_vec<-rep(0,R)

res1<-max_cor(X,T,PHI)
testhat<-res1$Maximal_cor

out <- meboot(x=X, reps=R, trim=0.10, elaps=TRUE)

for(i in 1:R){
res2<-max_cor(out$ensemble[,i],T,PHI)
cor_vec[i]<-res2$Maximal_cor
phase_vec[i]<-res2$Maximal_cor_peakTime
}

pval <- (sum(cor_vec >= testhat)+1)/(R+1)

res<-list(phase_vec=phase_vec,pval=pval)

return(res)
}

#==============================Multiple meboot analysis==============================
# The following function do the whole analysis for whole data set

multiple_meboot_analysis<-function(data,R,T,PHI){

library(meboot)
p<-dim(data)[1]
q<-dim(data)[2]
phase_mat<-matrix(0,nr=p,nc=R)
quantile_mat<-matrix(0,nr=p,nc=2)
pval_vec<-rep(0,p)

for(i in 1:p){
res<-single_meboot_analysis(data[i,2:13],R,T,PHI)
phase_mat[i,]<-res$phase_vec
pval_vec[i]<-res$pval
}

boot_quan<-apply(phase_mat,1,quantiles_calc)
```

```
for(i in 1:p){
quantile_mat[i,1]<-boot_quan[1,i]
quantile_mat[i,2]<-boot_quan[2,i]
}

results<-list(phase_mat=phase_mat,
              quantile_mat=quantile_mat,pval_vec=pval_vec)

return(results)

}

#============Example: Run the method on the IWAT data====================
r1<-multiple_meboot_analysis(data=IWAT,R=999,T=seq(0,44,by=4),
                             PHI=c(0,pi/3,2*pi/3,pi,4*pi/3,5*pi/3))
```
